# National Public Health Institutes Organizational Strengthening Module Framework: Applying Organizational Development Principles

**DOI:** 10.5334/aogh.4994

**Published:** 2026-05-22

**Authors:** Bhakti Hansoti, Catherine Stodola, Smriti Ridhi, Binita Adhikari, Cyrus Engineer, Shelly Bratton, Caroline Carnevale, Julius S.M. Gilayeneh, Roma Chilengi, Sara Bennett

**Affiliations:** 1 Department of Emergency Medicine, Johns Hopkins University, Baltimore, MD, USA; 2 Department of International Health, Johns Hopkins Bloomberg School of Public Health, Baltimore, MD, USA; 3 Division of Global Health Protection, CDC, Atlanta, Georgia, USA; 4 National Public Health Institute of Liberia, Monrovia, Liberia; 5 Zambia National Public Health Institute, Lusaka, Zambia

**Keywords:** National Public Health Institutes, organizational strengthening, global health security, NPHIs

## Abstract

*Background:* National Public Health Institutes (NPHIs) are government agencies that provide science-based leadership and coordination for public health functions. They are often formed by merging existing public health entities and functions found across and beyond the health sector, seeking to reduce fragmentation and enhance national preparedness and response. Their effectiveness depends on scientific rigor and sustainable capacity that can endure fluctuations in funding and priorities.

*Objective:* To present the NPHI Organizational Strengthening Module (OSM) framework—a practical, adaptable set of tools designed to strengthen organizational design and performance management of NPHI’s to institutionalize core public health functions and improve resilience and responsiveness to emerging health threats.

*Methods:* The OSM framework originates from the field of organizational development (OD) and builds on approaches validated in business and public sectors. The framework is tailored to meet the specific OD needs of NPHIs and was developed through reviewing and adapting tools, combined with piloting of each module.

*Findings:* The OSM framework includes a foundational public health function mapping activity and has six independent modules: strategy, partnerships, communication, workforce, finance, and governance—supported by a cross-cutting change management workstream. Each OSM consists of 3–4 concrete steps, beginning with a domain-specific assessment, followed by guided actions and best practices. OSMs are delivered through 3–6-month sprints, typically culminating in a 2–5-year strategy. Designed to engage senior leadership, the tools can be implemented virtually or in-person by internal staff or external facilitators.

*Conclusions:* The OSM framework provides a systematic, evidence-informed approach to organizational strengthening for NPHIs. By focusing on strengthening the strategic and operational capacity of the organization, this approach supports NPHIs in becoming more effective and resilient stewards of national public health, capable of anticipating and responding to emerging health threats.

## Background

**Figure 1:**
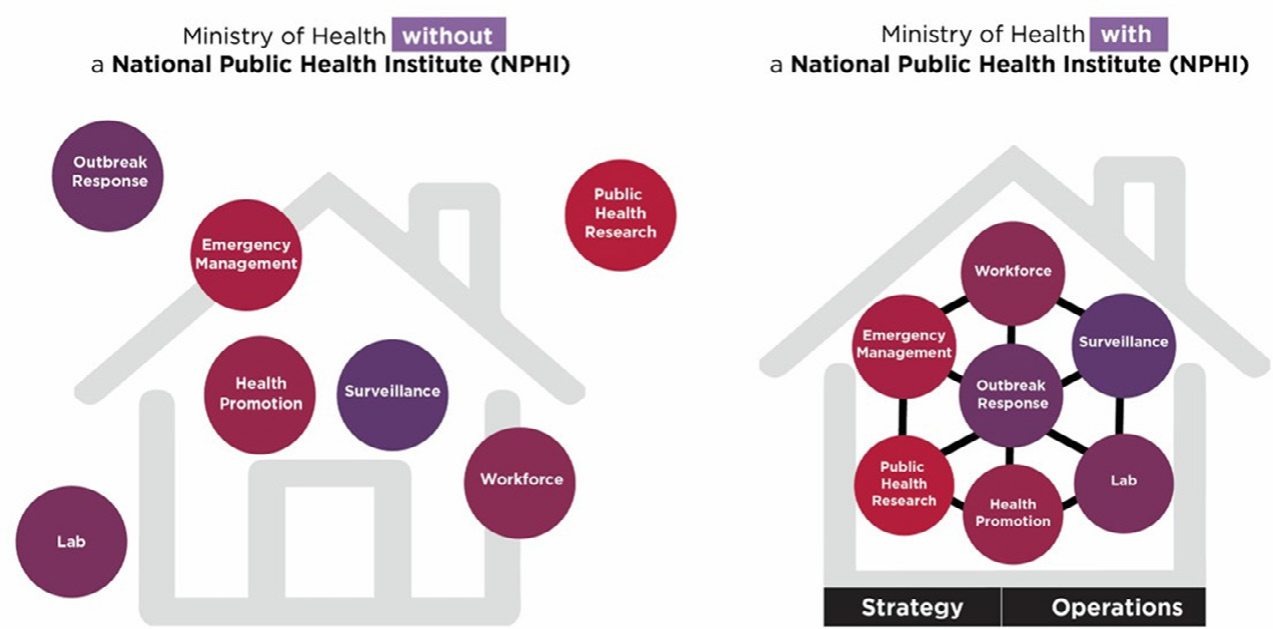
Improving linkages between public health functions and organizations: U.S. Centers for Disease Control and Prevention.

As the COVID-19 pandemic and other major public health events have highlighted, there is a growing need to strengthen and integrate public health systems [1–3]. Organizations such as the World Bank, World Health Organization (WHO), and International Association of National Public Health Institutes (IANPHI) supported the development of National Public Health Institutes (NPHIs) to strengthen core public health functions through technical leadership, enhanced governance and coordination, and improved national capacity to prevent, detect, and respond to public health threats [4, 5]. NPHIs are defined by IANPHI as “a government agency, or closely networked group of agencies, that provides science-based leadership, expertise, and coordination for a country’s public health activities” [6]. NPHIs are often established through the merging of existing public health entities or functions within a country’s Ministry of Health and relevant organizations from other sectors to reduce fragmentation and improve coordination of outbreak detection and response (Figure 1) [7]. NPHIs have been vital for the development of national preparedness and response plans; the rollout of COVID-19, mpox, and other emergency responses; workforce development; and addressing vaccine hesitancy [6, 8–13].

An NPHI’s core functions are “a set of fundamental activities that address the determinants of health, protect a population’s health, and treat disease” [14]. Adapted from the essential public health functions (EPHFs), they address population needs during times of peace, stability, and emergencies [15]. IANPHI highlights three functions for NPHIs: (1) evaluation and analysis of health status; (2) public health surveillance, investigation, and control of risks and threats; and (3) public health research [6, 16]. Each function is dynamic and requires stable organizational infrastructure to ensure consistent execution, effective coordination across systems, and the flexibility to adapt to evolving public health needs and emerging threats [17].

While some NPHIs have been established for decades, many countries have established NPHIs more recently in response to disease outbreaks such as SARS, Ebola, and COVID-19 [4, 10, 18, 19]. The exact structure, governance, and functions of NPHIs vary by country, but common themes of coordinating national public health responses and improving health security are consistent among all [2, 6, 10, 12, 18]. While there is a collective recognition that the delivery of public health functions requires focused attention on the capacity and management of public health organizations, there is little written on how this can be achieved [2, 4, 20]. Thus, NPHIs and global health security are vulnerable to changes in leadership, funding, political will, and emergency needs.

Two tools widely used to define country level health security capacity include the Joint External Evaluation (JEE) developed by the World Health Organization and partners, and the Staged Development Tool (SDT) developed by US CDC in partnership with IANPHI. The JEE focuses on country level capacity, uses a standardized scoring approach to assess capabilities across 19 technical areas, helps countries identify priorities for strengthening health security and supports country leaders to develop a National Action Plan for Health Security [21]. The JEE does not support countries in identifying organizational weaknesses or in tailoring strategies to address these gaps [21–23]. The SDT is designed to focus on NPHI capacities. It encompasses both internal domains (such as leadership and management, and staff development) as well as externally facing ones (such as surveillance and emergency preparedness) [24]. The tool is broad, encompassing 27 distinct domains [24, 25]. Self-assessments are conducted through discussion guides that enable participants to assess maturity of different systems, prioritize gaps, and develop a plan to address those gaps. Both of these tools assess maturity assessed along a continuum and the tools assume that NPHI leaders have the requisite capacity to identify root causes of problems [9].

The goal of our work was to develop a complementary approach that can systematically be applied to strengthen the organizational capacity of NPHIs to fulfill their core public health functions while remaining responsive to emerging health challenges. This article presents a framework and set of tools, grounded in performance-centered Organizational Development (OD) principles. OD is an evidence-based discipline focused on enhancing an organization’s capacity to manage change and improve performance in both current and future operations [26–28]. While traditionally applied in business and private-sector settings, OD principles have been adapted for use in the public health sector [29, 30]. By addressing the structure of roles, processes, and relationships within institutions, OD principles can support NPHIs in building the organizational and health system capacity needed to fulfill their public health mandates [31, 32].

In preparation for the work reported here, we conducted exploratory assessments with NPHI leaders, leadership within the US CDC’s Institutional Strengthening Team, along with a rapid scoping review to understand organizational development challenges commonly faced by NPHIs and how existing tools designed to support the OD of NPHIs were being used. This initial exploratory analysis revealed that (1) while tools for assessing OD components of NPHIs exist and are utilized, they typically focus on describing the status quo rather than helping NPHI leadership identify appropriate strategies to address the issues identified. Further, NPHI leaders argued that such tools and assessments must be responsive to local context. (2) While NPHIs function in unique contexts and are at different stages of maturity, there was a convergence in OD needs. Specifically, NPHIs, both more nascent and well-established ones, typically prioritized domains related to financial management and diversification; stakeholder engagement; workforce planning and development; and leadership training. (3) NPHIs need support to build OD capacity. As science-driven institutions tasked with leading and coordinating EPHFs for a country, training and capacity building efforts have usually focused on technical capacity, whereas we found a lack of skills on OD topics, such as workforce planning, staff development, strategic communications, or financial management.

This framework, the Organizational Strengthening Module (OSM) framework, was developed collaboratively by Johns Hopkins University (JHU) and the U.S. Centers for Disease Control and Prevention (US CDC) with input from several NPHIs globally. It is designed to serve NPHI leadership, staff, and the partners working with them. In contrast to the tools described above, the OSM framework addresses relatively few domains (eight), all of which are internal looking and the framework provides a structured approach with predefined questions, tools, and checklists, unique to each module. Further, the OSM framework aims to guide NPHIs through a series of analytical and diagnostic steps that are tailored to their own context, and links to concrete and relevant strategies to address identified gaps.

## Methods

We developed the OSM framework by adapting existing OD frameworks. Modules were sequentially designed and contextually adapted, informed by formative inquiry through stakeholder engagement, and then piloted across multiple countries using virtual and in-country approaches. This process took place between 2021–2025 with iterative refinement of the modules during piloting so as to ensure relevance to NPHI ecosystems, governance structures, and varying levels of institutional maturity.

### OD frameworks

The two main OD models that informed the OSM framework were the STAR model and the Baldrige Performance Excellence Framework [33, 34], which are widely recognized organizational design and performance improvement tools intended for use by private, public, and nonprofit organizations.

The STAR model, developed by Jay R. Galbraith, is an *organizational design* model that defines five critical and interrelated categories: strategy, structure, processes, rewards, and people. Strategy is seen as the primary driver of other aspects of organizational design, and Galbraith argues that to become a high performing organization it is most critical that these five aspects of the organization are aligned [34, 35]. Strategy concerns the organization’s directions and goals, as set out in its strategic plan. Structure concerns where decision-making power lies in an organization, and processes address how information flows within and outside of an organization [35]. While distinct, the categories of people and rewards both concern an organization’s workforce planning efforts as they focus on the skills and motivation of employees, respectively [35]. Given the mandate of NPHIs to lead and coordinate public health functions within a country, the model’s emphasis on “form follows function” and that an organization’s structure should be based upon and reflect the functions that it is supposed to assume became an essential element of framework development. The STAR model’s categories also resonated with the themes heard from NPHI leadership regarding the importance of aligning strategic planning, strategic communications, and workforce development.

The Baldrige Performance Excellence Framework is an OD model with a comprehensive and validated set of assessment criteria developed by the Baldrige Performance Excellence Program to guide organizations to performance excellence [33]. The seven categories of the framework are organized into two interlocking triads: leadership and results [33, 36]. The leadership triad connects leadership with the strategy and customer categories, as leadership is key for setting an organization’s strategic objectives, vision, and ongoing success [33, 36]. The results triad connects results with workforce and operations as a means to drive the production of services [33, 36]. Baldrige’s system perspective resonated with what was heard from NPHIs as they desired flexible tools that addressed contextual nuances anchored in objective and leadership-driven solutions to OD issues.

The team adapted and merged these models to arrive at an overarching framework, tailored to the priority needs of NPHIs.

### Tool development and adaptation

The modules in the OSM were developed sequentially, beginning with foundational modules and progressively building toward more advanced areas. For each module, we aimed to identify two pilot NPHIs to test and refine the approach. Development of each module began with a scoping exercise to identify existing tools for assessing organizational capacities within the specified domain. Early meetings with NPHI leaders and teams where the tool was to be piloted shed light on the specific challenges they were facing and focused our exploration of how the modules could support their needs. Insights from these discussions shaped the objectives of each module and informed the selection of tools used to deliver them. In most cases, tools required adaptation to ensure they were relevant to the NPHI context. These adaptations often involved accounting for the complex ecosystem of actors that NPHIs engage with—including both domestic and international partners, as well as health and non-health sector stakeholders—and modifying financing tools to reflect the nonprofit status of NPHIs and their reliance on government funding. Additional adjustments were also made to reflect varying levels of institutional maturity, governance arrangements within public sector systems, and the need to align tools with existing national public health strategies and planning processes.

### Piloting

The OSM framework and tools were piloted in four countries that requested support from the CDC Institutional Strengthening Team (see Table 1). Countries were selected for piloting based upon an alignment between their interests in OD and particular OSMs that the team were developing. Also, in some cases the team developed rapport with particular NPHIs that permitted the piloting of multiple different modules.

**Table 1:** Piloting of OSM framework and tools to date.

OSM	TIME FRAME OF ENGAGEMENT	MODALITY
Zambia—ZNPHI		
Assessment and Mapping of Public Health Functions	August 2021 to November 2021	Series of virtual Zoom meetings
Partnership Mapping and Strategy Development	September 2022 to January 2023	Series of virtual Zoom meetings In-Country Workshop
Liberia—NPHIL		
Strategic Planning	August 2021 to November 2021	In-Country Workshop
Workforce Strategy and Development	December 2022 to June 2023	Series of virtual Zoom meetings In-Country Workshop
Financial Strategy and Diversification	December 2023 to April 2024	Series of virtual Zoom meetings In-Country Workshop
Strategic Communications	March 2025 to June 2025	Series of virtual Zoom meetings
Sierra Leone—Nascent NPHA		
Strategic Planning	February to April 2021	In-Country Workshop
Burkina Faso		
Financial Strategy and Diversification	December 2023 to April 2024	Series of virtual Zoom meetings In-Country Workshop

Piloting typically occurred over a series of months, including both online and in-person modalities. The team took process notes throughout the engagement documenting what was and was not working. Frequent course adjustments were made during the process of module implementation, for example to bring a sharper focus to critical OD challenges or simplify tools for use in the field. Upon completion of a pilot, the team took stock of what did and did not work and made adjustments to the approach and tools used.

## Findings

### Framework for NPHI strengthening

**Figure 2:**
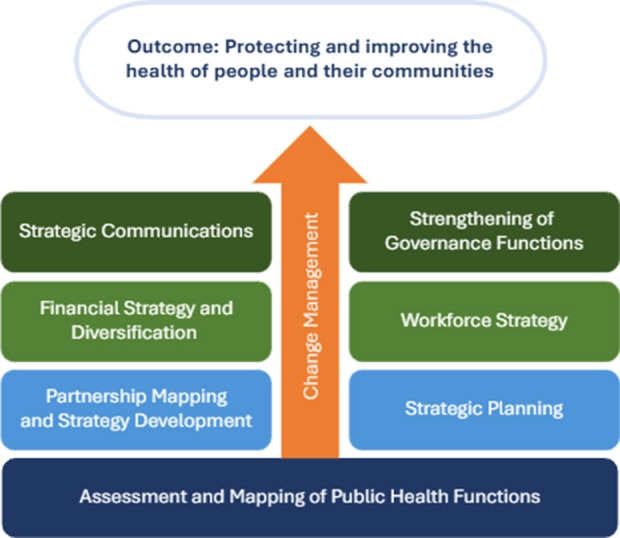
Framework for NPHI strengthening.

The OSM framework is composed of eight organizational strengthening modules, each focused on one priority domain for NPHIs (Figure 2). The dark blue base of the framework addresses the foundational question of what functions the NPHI is responsible for. Moving up the framework, there are two lighter blue OSMs focused on strategy and partnerships. These are also fundamental: the strategic planning OSM enables an NPHI to set out its vision, mission, goals, and strategies that in turn drive other organizational aspects. Partnerships are also fundamental because in many cases NPHIs need to work closely with other partners to deliver core functions, and from the needs assessment, we found that there was frequently confusion around NPHI roles versus those of partners. The next two pale green OSMs speak to core organizational systems, namely workforce and financing, while the two dark green OSMs at the top of the figure address higher level concerns, notably governance and strategic communications. Running through the center of the figure in orange is a focus on change management, reflecting the importance of enabling NPHIs to transition to new organizational arrangements smoothly, minimizing disruptions to the services they provide. In practice, change management concepts are built into each of the other modules.

### Organizational strengthening modules

Each OSM is made up of 3–4 concrete steps, beginning with a domain-specific structured assessment followed by additional steps that facilitate reflection and provide guidance on best practices and strategies for NPHIs to achieve desired OD milestones. Once completed, each OSM produces an actionable strategy that can be feasibly implemented over the next 2–5 years. While each module focuses on a discrete OD domain, all work contributes toward the outcome of independently and sustainably strengthening an NPHIs’ ability to protect and improve the health of the people and communities whom they serve through organizational strengthening.

The OSM *Assessment and Mapping of Public Health Functions* serves as an anchor for determining, prioritizing, and communicating the organizational priorities of NPHIs. It reflects the principles of Galbraith’s STAR model that form follows function and ensures that before other OSMs are addressed there is a clear understanding of the public health functions that the NPHI is intended to perform. While the completion of the SDT often serves as a helpful first step to ground the discussion in current reality, this OSM goes substantially further identifying specific functions and mapping the role of the NPHI vis a vis other organizations in the delivery of these functions. The first step in the OSM utilizes Decision Space Analysis to identify the current decision-makers for various NPHI functions, determining where there is a gap or where many people/organizations are responsible for a given function to identify opportunities for growth or greater efficiency. Process mapping is then used as the second step, building off of the Decision Space Analysis activity, to visually represent workflows and decision points, and to identify system bottlenecks. These bottlenecks then feed into the third step, root cause analysis, to tease out the most common and actionable underlying causes of the problem and facilitate identification of areas for change within the NPHI. Finally, step four is a prioritization activity that helps the NPHI identify other OSMs most relevant to its needs.

The *Strategic Planning* OSM is a logical next step after an NPHI’s core public health functions are mapped. Broadly, strategic planning encompasses strategy development, implementation, and the measurement of progress toward objectives. The OSM integrates key principles of both Baldrige and Galbraith’s STAR models to highlight tools that support NPHIs in the strategy development process. Activities include conducting an environmental scan of the NPHI’s current state to understand the NPHI’s strengths, weaknesses, opportunities, and threats along with what, if any, strategic planning activities have been completed. The OSM then offers guidance on drafting key elements of the strategic plan providing examples of mission, vision, core values, and goal(s) statements from similar entities, and providing a pathway for the NPHI to tailor these to their own needs. Finally, guidance on identifying strategic objectives and strategies to achieve these are provided, leaving the NPHI with a comprehensive draft strategic plan for further refinement.

Building the capacity of NPHIs to both develop and effectively manage stakeholder relationships was a commonly cited OD need. Establishing and maintaining partnerships is complex and requires an intentional approach driven by NPHI leadership given how priorities, operations, and funding models differ between partner organizations. The *Partnership Mapping and Strategy Development*OSM helps NPHIs review a broad swathe of stakeholders, identify key partners, and integrate the needs and interests of different local and international partners that expect services from the NPHI, while being strategic about growth and success by identifying opportunities for new partnerships and priorities. Based on concepts of social network analysis, OSM activities focus on using the NetMap tool to determine key linkages between an NPHI and its stakeholders so that NPHIs can better understand stakeholder goals, reasons for investment, and assess the ability of a stakeholder to influence the NPHI’s work [37].

Identified by both Baldrige and Galbraith’s STAR model as a key category to consider in terms of OD and design, and cited as a key issue by NPHI leadership, the *Workforce Strategy* OSM focuses on how NPHIs can build a competent and capable public health workforce for the NPHI, able to carry out routine and emergency public health functions. The OSM integrates workforce planning approaches with engagement strategies to guide an NPHI toward planning a fit-for-purpose workforce while also building an organizational culture that motivates and retains talent. OSM activities focus on conducting a workforce analysis (including assessing workforce supply, demand, and gap analysis) so that NPHI leadership can understand the current state of the NPHI’s workforce, forecast workforce needs, and identify and prioritize gaps to reach the NPHI’s desired workforce state.

NPHIs typically depend on a relatively narrow range of funding sources that could expose them to significant vulnerabilities, hinder sustainability, and lead to challenges in delivering their strategic plan. This dependency can also reduce flexibility in resource allocation and leave NPHIs constrained in their ability to adapt and respond effectively to public health emergencies. The *Financial Strategy and Diversification* OSM is meant to address some of these concerns, providing tools for an NPHI to strengthen operational autonomy, sustainability, and execution of financial functions. The first step in the OSM is a comprehensive financial health diagnostic assessment to review the NPHI’s current financial infrastructure, revenue streams, and overall financial needs and approach so that opportunities for improvement and strategies can be identified to draft a financial strategy. Tools and guidance on soliciting additional support from Ministries of Finance and development partners are provided.

The *Strengthening of Governance Functions* OSM focuses on how strong internal regulatory and administrative functions help protect an NPHI’s scientific independence and credibility along with ensuring clear lines of accountability within organizational structures. The OSM focuses on reviewing the current accountability structures of an NPHI. The NPHI maps its financial performance and political accountabilities using an accountability matrix to consider where these may need to be strengthened [38]. In addition, the OSM reviews governing and advisory board arrangements for the NPHI and uses validated approaches for both the assessment and strengthening of advisory or governing boards to ensure the effective operations of these entities [39–41].

Communicating the right message, to the right audience, at the right time is key for NPHIs to build credibility with the public, funders, and other stakeholders. The *Strategic Communications* OSM focuses on this concept and responds to NPHIs’ identified needs to communicate their mission, advocate more effectively for legal establishment or increased support, and navigate conversations in complex political environments.

Central to the framework is an OSM on *Change Management*; managing change effectively is crucial to being able to implement any of the other OSMs as well as to the successful establishment of a new NPHI. The OSM is at the center of the framework as it can be either utilized alone to develop a broad change management strategy (for example, for a new NPHI) or used in concert with any of the OSMs as an implementation tool. The Change Management OSM utilizes John Kotter’s Leading Change methodology, walking NPHIs through the validated eight-step framework to learn how to identify, clearly articulate, disseminate, and sustain the desired lasting change within the NPHI [42].

Table 2 provides additional information on the goal, objectives, suggested activities, and key outputs of each OSM.

**Table 2:** OSM goals, activities, and key outputs.

ORGANIZATIONAL STRENGTHENING MODULE (OSM)	GOAL	ACTIVITIES	KEY OUTPUTS
Assessment and Mapping of Public Health Functions	Guide NPHIs to reflect on and strengthen their role in performing the public health functions outlined in their legal mandate and/or strategic plan.	Decision Space Analysis Process mapping building off DSA to identify bottlenecks in processes experienced by NPHI Root Cause Analysis taking process map bottlenecks OSM prioritization exercise	Completed decision space analysis matrix for 2–3 functions Process map for 2–3 functional units Completed root cause analysis for identified bottlenecks Prioritized OSMs for future action
Strategic Planning	Enable NPHI leadership to actively engage in the strategic planning process by introducing established strategic planning tools to the local context and fostering country ownership.	Conduct environmental scan (SWOT) Draft mission, vision, and values Draft goals and strategic objectives	Established and/or clarified the mission and vision of the NPHI Completed SWOT analysis Strategic objectives for 3–5 years Draft NPHI strategic plan
Partnership Mapping and Strategy Development	Enable the NPHI to identify and prioritize key partners and provide support in developing a partnership strategy that addresses challenges and guides partner engagement.	Conduct social network analysis (NetMap) Partner presentations and/or discussions Examine partner influence and investment Develop partnership action plan	Network map of current and potential NPHI partners Draft partnership action plan
Workforce Strategy and Development	Develop guidance, tools, and workforce planning approaches to analyze, attract, retain, and nurture talent to meet current and future public health needs to effectively deliver core NPHI functions.	Determining strategic direction Supply analysis Demand analysis Gap analysis Culture and core values	Completed supply, demand, and gap analysis for NPHI workforce Demographic, capability, engagement, satisfaction, and burnout data for NPHI Draft workforce strategic action plan
Financial Management and Diversification	Develop a 3–5-year financial strategy with a focus on strengthening operational autonomy, sustainability, and execution of prioritized NPHI public health functions, as articulated in the organizational strategic plan.	Conduct NPHI financial health diagnostic assessment Identify opportunities for improvement Identify strategies and activities Develop action plan	Draft financial strategy
Change Management	Guide NPHI leadership through the fundamentals of change management to be able to understand and develop a comprehensive change management plan for the NPHI’s needs.	Articulate “burning platform” for change Form change coalition Develop change vision and communication plan Action plan for change	Draft change management strategy (broad, i.e., establishing a new NPHI) Draft change management strategy for OSM implementation (i.e., rolling out new strategic plan or financial management software, etc.)
Strengthening of Governance Functions	Support the establishment and/or strengthening of NPHI governance functions to protect the scientific independence, credibility, and agility of NPHIs.	Review of current governance structures Identify current and future governance needs Define barriers and resources needed to establish or strengthen governance structures and develop a plan to address them Develop strategic guidance for establishing new governance structures as needed by the NPHI (i.e., board, advisory board, audit unit)	Standard operating procedures for various processes
Strategic Communications	Equip NPHIs with the necessary knowledge, skills, and strategies to develop a plan in which they can effectively communicate with various stakeholders.	Review fundamentals of strategic communications Identify communication goal(s), target audience(s), objective(s), and key messages Review key communication strategies applicable to NPHIs Draft Strategic Communications plan Provide communication strategy toolkit	Draft strategic communications plan

### Implementation of organizational strengthening modules

While there are strong connections and substantial synergies between the OSMs, each OSM is designed to be a standalone set of tools, meaning that users can employ an OSM on its own or in concert with others. It is recommended, however, that the Assessment and Mapping of Public Health Functions OSM be conducted as an initial step, as it allows users to better understand the functions of the NPHI and helps define priorities across other OSMs.

Tool materials include facilitation guides, worksheets, presentations, case studies, and suggested readings all of which are tailored to the needs of NPHIs and presented in a format that allows users to edit easily for different contexts. While it is recommended that all the tools and activities presented in each OSM are used, there is guidance throughout stating where users can modify or skip activities.

Each OSM is designed to be implemented in a sprint, which is short and defined time periods of 3–6 months. Typically, a team of 3–4 NPHI staff members will serve as OSM leaders. The OSM team meets on a regular basis (either virtually or in-person) to collect relevant organizational information, review materials, and prepare for an in-country workshop. The workshop often represents the culmination of work where information is reviewed, strategies are refined, and plans for next steps are put in place. Such workshops also provide an opportunity to collect the perspectives of a broader array of stakeholders and secure buy in to the process.

### Piloting

Given the extensive piloting conducted, it is challenging to summarize succinctly key findings from this process; however, we offer insights from two sets of pilots namely the application of (i) the partnership module with ZNPHI in Zambia and (ii) the workforce module in Liberia, as a means of conveying the nature of learnings from pilots, before pivoting to cross-cutting lessons.

ZNPHI wished to implement the partnership module as it recognized that it had a multitude of domestic and international partners, who did not always clearly understand the role of ZNPHI, and managing these partners and their interests across multiple different technical functions was a significant burden on ZNPHI staff, and also led to role confusion and shifting priorities. Online engagement of the ZNPHI team tasked with taking forward this OSM led to an initial listing of stakeholders and an articulation of key challenges, as well as objectives for what the institute wanted to achieve through the module. The in-person workshop was designed to enable ZNPHI staff to reflect on what they needed from partners and how they could be more effectively managed, and also bring in a diversity of partners to understand their perspectives. The workshop led to several concrete outcomes, including a more refined categorization of partners, a set of possible strategies for managing partners (from broad all partner communications to more targeted strategic communications with individual partners), as well as a draft partner engagement strategy. Insights from the discussion informed OSM slide decks and templates, enabling other NPHIs to benefit from ZNPHI’s experience. From ZNPHI leadership’s perspective, the workshop created a valuable space to reflect on partnership effectiveness, strengthened ownership of partnership management, and reinforced the importance of at times saying “no” to partners.

NPHIL’s strategic plan identified the critical lack of trained and motivated public health workers in Liberia as a key constraint facing the institute. Accordingly, NPHIL’s leadership was keen to apply the Workforce Strategy and Development module. Leading up to the workshop, the JHU team supported NPHIL in conducting a survey of its existing staff that assessed staff characteristics, pre-service and in-service training, roles, and workforce engagement and satisfaction. During the workshop, NPHIL staff reviewed survey findings and discussed priority concerns and what could be done about them. This led to an important shift in priorities. The NPHIL team recognized that with funding constraints it was not possible to hire more skilled staff or conduct extensive training for existing staff, but there were other levers within their grasp such as strengthening orientation for new staff, building clearer career pathways to support promotion, introducing mechanisms for staff engagement, and developing a human resource information system that could all help achieve workforce goals. This shift in perception underscored the importance of purposively building the evidence base, staying open-minded to alternative perspectives, and using group processes to establish priorities.

Across the pilots, several implementation lessons emerged. First, inclusive participation—engaging both senior leadership and technical leads across core public health functions—was essential for accurate functional mapping and alignment with operational realities. For example, mapping exercises showed that functions such as surveillance are dynamic and differ across preparedness and response phases, requiring flexible, phase-sensitive maps and cross-functional comparison. Second, strategic planning was most effective with active leadership engagement; in Liberia, limited ownership of existing strategic plans underscored the need to realign strategic priorities with actual work, with leadership involvement enabling function leads to drive that realignment. Third, modules were interdependent—experience in Zambia revealed an overwhelming and diverse partner landscape, highlighting the need to pair partnership mapping with a strategic communication approach and reinforcing the importance of sequencing modules. Fourth, while virtual, hybrid, and in-person modalities were feasible, in-person engagement was particularly valuable for newer NPHIs, such as in Sierra Leone, where physically convening leadership supported alignment, trust-building, and shared understanding; cultural preferences also shaped modality effectiveness. Across sites, an approximate two-month time frame per module balanced time for reflection, data gathering, and consensus-building without prolonging implementation and risking attrition due to competing priorities. Finally, sustainability was strengthened by identifying local champions to co-lead, who would build familiarity with the methodology, and carry forward the work, particularly in settings with high leadership turnover.

## Discussion

We have reported on the design, piloting, and implementation of a new framework and set of tools to support the OD of NPHIs. While other frameworks and tools are available, they are not strongly focused on OD or supportive of developing plans to address OD challenges. Our approach to develop these OSMs sought to adapt well-established OD frameworks and tools so that they met the specific needs of NPHIs. Here, we reflect on our learnings from the process of developing and testing the OSMs and the insights they offer for future implementation and scale-up of the OSMs.

First, leadership engagement was key to success across countries. In those contexts where senior and technical leaders engaged with the OSMs, there was the greatest impact. While the OSMs are focused on discrete functions, they adopt a strategic perspective, thus leadership engagement is needed to ensure a commitment to change and accountability for completing the work and implementing recommendations, and a broader vision for the work is established. NPHIs with empowered focal points for the OSMs had greater consistency in leadership and staff engagement, as well as better follow-through in application of the OSMs. In addition, a mix of technical and operational staff provided a holistic understanding of the topics covered in each OSM and developed recommendations geared toward enhanced technical implementation considering the realities of operations.

In some contexts where the OSMs were piloted, turnover and shifting priorities disrupted continuity and required renewed alignment of efforts, leading to longer engagement timelines, inconsistent inputs, and patchwork outputs. Implementation was also challenged by competing urgent public health demands, virtual connectivity issues, and both financial and human capacity constraints. Many NPHIs had limited staffing, so competing demands such as outbreaks or other routine activities could disrupt timely progress and consistent engagement. This underscores the need for flexible implementation timelines, continued coordination with programmatic leads, and strategies to institutionalize OSM workstreams amid a dynamic operating environment. An approach to this is to make sure that the domains covered in the OSMs are relevant to current challenges. For example, in Zambia the partnership mapping OSM was purposively focused on Global Health Security as an urgent priority area. Sequencing OSMs in response to institutional priorities and readiness appeared to foster stronger engagement and greater ownership. This points to the importance of using a demand-driven and context-sensitive approach when rolling out OSMs.

One innovation incorporated during implementation was having discrete engagements with clear deliverables, where remote connection was used pre- and post- workshops to ensure that activities were completed and momentum was maintained. Each step built on the previous one, requiring flexibility to adapt based on emerging findings. This approach aligns with agile project management and is commonly referred to as “sprints”—referring to short, focused periods of intensive work aimed at achieving specific objectives or completing tasks within a limited time frame [43, 44]. Sprints allow for speed, flexibility, and enhanced focus, which is important in an ever-evolving landscape [45].

While not primarily a training tool, the OSMs integrate leadership development to ensure participants understand both the sequence of activities and the underlying organizational development principles, fostering appropriate engagement and ownership among NPHI leaders. We found that these approaches introduced a new lexicon for many NPHI staff, and while leaders and staff throughout the NPHI had extensive training on the medical and public-health-related elements of their job, many of the OD approaches shared were new. Training leaders to understand OD concepts, in particular change management, is essential to ensuring sustained success. With a deeper understanding of the process, leaders are able to take ownership of the process and ultimately the strategies developed.

To date there has been relatively limited application of these OSMs. Continued use by NPHI leadership and staff, US CDC staff, and other partners involved in NPHI strengthening will add to the body of knowledge and help strengthen the OSMs in the process. While facilitation guidelines are included within the OSMs, we reflect that it may sometimes be intimidating for people without a background in OD to use these tools. Ideally, cohorts of facilitators and consultants would be trained to work with these OSMs to support NPHI OD.

## Conclusion

NPHIs are often the preeminent organizations responsible for protecting and promoting the public’s health, especially in the face of communicable disease outbreaks. While intensive effort has been invested in enhancing the technical capacities of NPHIs, inefficient organizational structures and processes can impede technical excellence. The OSM framework presented here is the first tool to focus primarily on NPHI organizational strengthening, providing tailored assessments and solution strategies. Preliminary reflections underscore the paramount need for strong leadership in the success of these initiatives, while the flexibility and adaptability of OSMs are crucial to accommodate the competing demands on NPHI staff. Using validated OD tools to provide a structured framework to strengthen organization design and performance management will empower NPHIs to respond more efficiently and effectively to public health challenges, enhancing global health security and improving public health outcomes globally.

## Additional File

The additional file for this article can be found as follows:

Supplementary Annex 1. PRISMA Diagram for the peer-reviewed and grey literature.
