# Supplementary material for: National Public Health Institutes Organizational Strengthening Module Framework: Applying Organizational Development Principles
Source: Ann Glob Health. 2026 May 22;92(1):45. doi: 10.5334/aogh.4994 (PMC13196681; doi:10.5334/aogh.4994)

Annex 1.

PRISMA Diagram for the peer-reviewed and grey literature

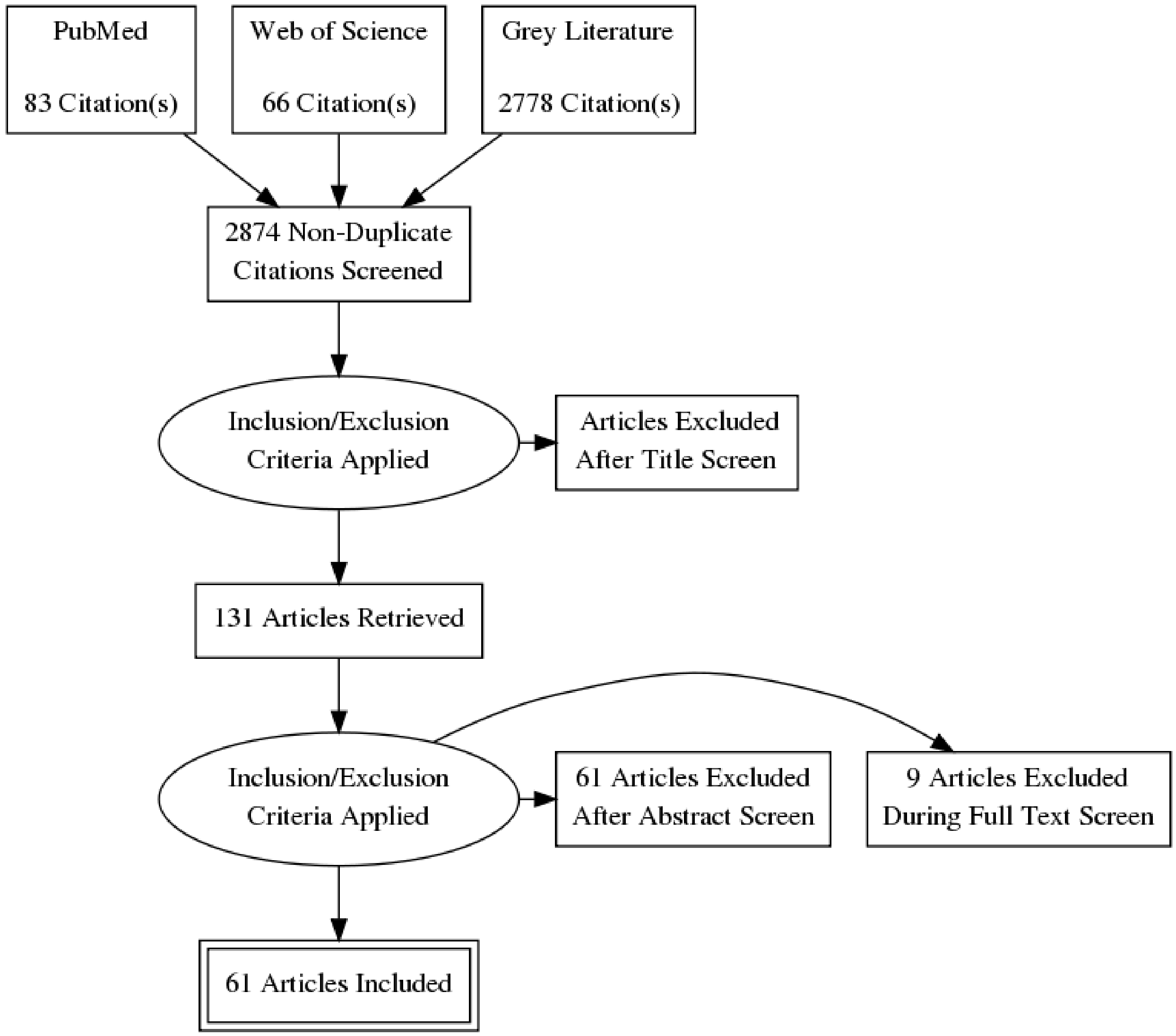

Supplement: Supplementary Annex 1. — PRISMA Diagram for the peer-reviewed and grey literature. [file agh-92-1-4994-s1.pdf]
